# Protein in nutritional support: the newborn hero for the critically ill?

**DOI:** 10.1186/s13054-014-0592-z

**Published:** 2014-11-17

**Authors:** Taku Oshima, Claudia P Heidegger, Claude Pichard

**Affiliations:** Nutrition Unit, Geneva University Hospital, Geneva 14, 1211 Switzerland; Department of Anaesthesiology, Pharmacology and Intensive Care, Geneva University Hospital, Geneva 14, 1211 Switzerland

## Abstract

In their current review, Weijs and colleagues highlight the importance of protein and amino acid provision for improving clinical outcome in critically ill patients. The interdependence between energy and protein is highlighted. They call for urgent research to develop new methods to evaluate protein and amino acid requirements, accurately and conveniently, in order to optimize nutrition support for critically ill patients.

Appropriate nutrition delivery for critically ill patients remains a highly debated issue. Energy, a critical factor for life, was until now the superstar of nutrition support. It now faces a rival or, more correctly, a partner in function, namely protein. This is a chance to take a close look at protein, the new hero in the field of critical care nutrition, and the struggles it encounters in becoming the true superstar.

In their current review, Weijs and colleagues [1] highlight the importance of protein and amino acid provision for critically ill patients, the combination of which for adequate energy provision has been shown by an increasing number of studies.

Providing nutrition support for critically ill patients is generally recommended as a standard of care. This approach is confirmed by recent studies which demonstrated the benefits of early and adequate nutrition support, whenever possible by the enteral route. The nutrition target is often defined as calculated calories/kg body weight per day, or measured by indirect calorimetry. But what about the protein target and its measurement?

Disturbances of protein metabolism are observed as a physiologic response to stress, and are reflected by important nitrogen loss and muscle wasting which are proportional to the severity of illness. Amino acid administration acts as a main signal for protein synthesis in healthy conditions, but does not reduce protein breakdown [2]. This signal is blunted in critically ill patients due to anabolic resistance [3]. Ishibashi and colleagues [4] suggested that protein catabolism over a 10-day period soon after ICU admission was reduced by 50% when protein intake increased and allowed a reduction of muscle loss of 1.1 to 1.5 g/kg of dry fat-free mass/day (that is, lean tissue without water content). A higher level of protein intake (1.9 g/kg/day) did not confer any additional protein-sparing advantage, and 1.2 g/kg/day was suggested as the optimum dose. These suggestions for protein intake level of at least 1.2 g/kg/day are in accordance with the current recommendations [5,6]. On the other hand, a previous study by Weijs and colleagues [7] suggested that protein intake of 1.5 g/kg/day improves clinical outcome. Indeed, a number of contradictory studies have resulted in variations of the recommended protein provision over the past decades. In the 1970s, provision as high as 3.5 g/kg was recommended [8], but this was then reduced to 1.2 g/kg/day by 2006 [9]. During the same period, the recommended provision of calories has decreased from 40 to 45 to 20 to 30 kcal/kg/day. It is likely that such a decrease in energy administration has an impact on protein needs, because energy deficit increases proteolysis to fuel obligatory gluconeogenesis. Currently, there is a trend among experts to increase daily protein provision up to 1.5 to 1.8 g/kg/day, and to reconsider the protein/energy ratio to optimize the respective amounts of protein and energy administered [10]. Robust data are still lacking to support final recommendations. The importance of both components was shown recently by Weijs and colleagues [7], demonstrating that the achievement of defined protein and energy targets was associated with a 50% decrease in 28-day mortality in mechanically ventilated patients, whereas reaching only the energy target did not impact mortality.

Apart from highly contributing to the gross mass of body protein, amino acids are signaling molecules in a number of cellular processes, including those related to stress-related catabolism. This implies that not only the amount of amino acid but also their composition profile is important. It should be recalled that muscle wasting and protein loss during the acute phase of critical illnesses could only be mitigated by adequate and optimal supplementation of amino acids, along with energy and micronutrients.

Optimal nutrition for critically ill patients through the enteral route, though it is strongly recommended in recent guidelines, is difficult in some patients as gastrointestinal tolerance limits the rate of administration and absorption. For instance, diarrhea is commonly observed in critically ill patients and strongly associated with antibiotics, as well as with enteral nutrition if covering more than 60% of energy needs [11]. This frequently limits the prescription of full enteral nutrition support. In such cases, exclusive parenteral nutrition [12], supplemental parenteral nutrition [13] or a specific administration of amino acids to supplement enterally administered protein may be a therapeutic option. Nutrients administered through the enteral route must be digested, a process which increases the energy and oxygen demand of the intestine, which in turn increases splanchnic blood flow [14]. This effect may not be tolerated by very hemodynamically unstable patients.

The recommendations for protein provision in guidelines [5,6] are derived from expert opinion, rather than from robust scientific evidence. This situation mostly results from the complexity and the costs of measuring protein synthesis and degradation, and the current lack of analytical methods that can obtain results sufficiently rapidly to enable the prescription of nutrition support to be altered appropriately. Weijs and colleagues give a great overview of the complexity involved in assessing protein and specific amino acid requirements, and point out the interdependence between energy and protein provision. We join their call for urgent research to develop new methods to evaluate protein and amino acid requirements, accurately and conveniently, in order to optimize nutrition support for critically ill patients.
